# Supplementary material for: Virtual Screening, Synthesis, and Biological Evaluation of Some Carbohydrazide Derivatives as Potential DPP-IV Inhibitors
Source: Molecules. 2022 Dec 24;28(1):149. doi: 10.3390/molecules28010149 (PMC9822036; doi:10.3390/molecules28010149)
Supplement: Supplementary file 1 [file molecules-28-00149-s001.zip › molecules-2018650-SI.pdf]

Supplementary File

Table S1. The binding interactions of the molecules with the target enzyme

Active amino acid residues	Atom from ligand	Bond length (Å)	Bond type	Bond category	Ligand energy (kcal/mol)	Docking scores (kcal/mol)
			1a			
**LYS71**		4.96902	Electrostatic	Pi-Cation		
**PHE95**		4.92287		Pi-Pi T-shaped		
**ILE102**		4.94969				
**LYS71**	Pi-Orbitals	5.35011	Hydrophobic		288.14	–8.9
**LEU90**		5.37861		Pi-Alkyl		
**ILE102**		5.21468				
			1c			
**PRO510**	H	2.22317				
**ARG40**	O	2.03718		Conventional Hydrogen Bond		
**ARG40**	O	2.57182	Hydrogen Bond			
**PRO475**	C	3.62995		Carbon Hydrogen Bond	335.05	–8.8
**MET509**		3.59319		Pi-Sigma		
**LYS512**		3.89571				
**LEU504**	Pi-Orbitals	4.54261	Hydrophobic	Pi-Alkyl		
**MET509**		5.42387				
			1d			
**GLU378**	N	5.27299	Electrostatic	Attractive Charge		
**GLU378**	N	5.29489				
**ASP588**	H	2.41364	Hydrogen Bond			
**SER376**	H	2.12146		Conventional Hydrogen Bond		
**ATHR351**	O	2.50761	Hydrogen Bond		311.65	–8.9
**ILE346**	C	3.37113		Carbon Hydrogen Bond		
**ARG382**		4.58332	Electrostatic	Pi-Cation		
**ILE375**	Pi-Orbitals	5.17097	Hydrophobic	Pi-Alkyl		
**VAL354**		5.07038				
			2a			
**PHE95**	Pi-Orbitals	4.98113		Pi-Pi T-shaped		
**ILE102**	Cl	3.49641	Hydrophobic	Alkyl	339.38	–8.7
**ILE102**	Pi-Orbitals	4.88951		Pi-Alkyl		
**LEU90**		5.11987				
			2c			
**ILE107**	H	2.28309	Hydrogen Bond	Conventional Hydrogen Bond	378.97	–8.8
**TRP157**	NH	2.96701				

**ARG61**	O	2.40749				
**ILE107**	O	2.64522				
**TRP157**	O	2.06362				
**SER212**	C	3.77751		Carbon Hydrogen Bond		
**LEU214**	C	3.77116				
**SER106**	O	3.55398				
**SER106**	Pi-Orbitals	2.88245	Other	Pi-Lone Pair		
**TRP216**		5.49345	Hydrophobic	Pi-Pi T-shaped		
**PRO159**		5.20949		Pi-Alkyl		
			2d			
**ASN92**	H	3.0358	Hydrogen Bond	Conventional Hydrogen Bond	355.28	−8.8
**LYS71**	Pi-Orbitals	4.93727	Electrostatic	Pi-Cation		
**PHE95**		5.01659	Hydrophobic	Pi-Pi T-shaped		
**ILE102**	Cl	3.47487		Alkyl		
**ILE102**	Pi-Orbitals	4.99551		Pi-Alkyl		
**LYS71**		5.32536				
**LEU90**		5.21993				
			3a			
**GLU206**	H	2.41891	Hydrogen Bond	Conventional Hydrogen Bond	337.99	−8.8
**PHE357**	C	3.70779	Hydrophobic	Pi-Sigma		
**PHE357**	C	3.95473				
**PHE357**	Pi-Orbitals	3.99501		Pi-Pi Stacked		
**TYR547**		4.09098				
**PHE357**	Cl	4.71473		Pi-Alkyl		
**TYR547**	Cl	5.32992				
**TYR662**	Cl	4.47507				
**TYR666**	Cl	4.98485				
			3c			
**ILE107**	H	2.17441	Hydrogen Bond	Conventional Hydrogen Bond	374.16	−8.9
**THR156**	NH	2.0726				
**ARG61**	O	2.46899				
**ILE107**	O	2.51808				
**TRP157**	O	2.13552				
**SER106**	Pi-Orbitals	2.89668	Other	Pi-Lone Pair		
**TRP216**		5.44173	Hydrophobic	Pi-Pi T-shaped		
**PRO159**		5.1031		Pi-Alkyl		
**TRP154**	Cl	4.98516				
			3d			
**ASP545**	N	5.34106	Electrostatic	Attractive Charge	353.66	−8.8

**TYR752**	O	2.12135	Hydrogen Bond	Conventional Hydrogen Bond		
**TRP629**	Cl	3.8172	Hydrophobic	Pi-Sigma		
**TRP629**	Cl	3.98947				
**TYR547**	Pi-Orbitals	3.87254		Pi-Pi Stacked		
**TRP629**		4.19414				
**TRP629**		4.42556				
**LYS554**		5.44561		Pi-Alkyl		
**TRP629**	Cl	5.02642				
4b						
**ASN103**	H	3.07942	Hydrogen Bond	Conventional Hydrogen Bond	318.42	−8.9
**ILE102**	H	2.67703				
**HIS100**	H	2.06913				
**ILE102**	O	2.26069				
**ASN74**	F	3.08835	Halogen	Halogen (Fluorine)		
**LYS71**	Pi-Orbitals	4.96134	Electrostatic	Pi-Cation		
**TYR105**		5.0545	Hydrophobic	Pi-Pi T-shaped		
**ILE76**		5.40444		Pi-Alkyl		
**ILE102**		5.07332				
4c						
**ASN103**	C	3.57787	Hydrogen Bond	Carbon Hydrogen Bond	353.37	−8.8
**PHE95**	Pi-Orbitals	5.00945	Hydrophobic	Pi-Pi T-shaped		
**ILE102**		4.94343		Pi-Alkyl		
**LYS71**		5.38863				
**LEU90**		5.35004				
**ILE102**		5.31852				
4d						
**ASN92**	H	3.03667	Hydrogen Bond	Conventional Hydrogen Bond	330.06	−8.9
**PHE95**	Pi-Orbitals	4.96081	Hydrophobic	Pi-Pi T-shaped		
**ILE102**		4.99511		Pi-Alkyl		
**LYS71**		5.42747				
**LEU90**		5.30662				
**ILE102**		5.38307				
5a						
**PRO510**	H	2.08203	Hydrogen Bond	Conventional Hydrogen Bond	314.94	−8.8
**ARG40**	O	2.32727				
**ARG40**	O	2.4405				
**ASP501**	F	3.40107	Hydrogen Bond;Halogen	Carbon Hydrogen Bond;Halogen (Fluorine)		
**ASP501**	F	3.05778	Halogen	Halogen (Fluorine)		

**MET509**	Pi-Orbitals	3.63018	Hydrophobic	Pi-Sigma		
**PHE559**		5.66091		Pi-Pi Stacked		
**LEU504; GLN505**		4.85965		Amide-Pi Stacked		
**LYS512**		3.84082		Pi-Alkyl		
**LEU504**		4.6698				
**MET509**		5.10781				
5c						
**ASN103**	H	3.00917	Hydrogen Bond	Conventional Hydrogen Bond	352.31	−8.8
**ILE102**	H	2.29053				
**ASN92**	C	3.73613		Carbon Hydrogen Bond		
**ASN74**	F	3.09031	Halogen	Halogen (Fluorine)		
**TYR105**	Pi-Orbitals	5.14358	Hydrophobic	Pi-Pi T-shaped		
**ILE76**		5.49595		Pi-Alkyl		
**ILE102**		5.01132				
5d						
**GLU378**	N	5.1091	Electrostatic	Attractive Charge	330.02	−8.9
**GLU378**	N	5.11935				
**ASP588**	H	2.40892	Hydrogen Bond	Conventional Hydrogen Bond		
**SER376**	H	2.35939				
**THR351**	O	2.48898				
**GLY355**	F	1.95875	Hydrogen Bond;Halogen	Conventional Hydrogen Bond;Halogen (Fluorine)		
**ILE346**	C	3.3198	Hydrogen Bond	Carbon Hydrogen Bond		
**HIS592**	F	3.15587				
**GLU347**	F	2.7357	Halogen	Halogen (Fluorine)		
**TRP353**	F	3.68392				
**VAL354**	F	3.56747				
**ASP588**	F	3.06595				
**ILE375**	Pi-Orbitals	5.27418	Hydrophobic	Pi-Alkyl		
**VAL354**		5.26117				

Table S2. 2D- and 3D-binding postures of the molecules

3D Docking Pose	2D Docking Pose
Compound **1a**	
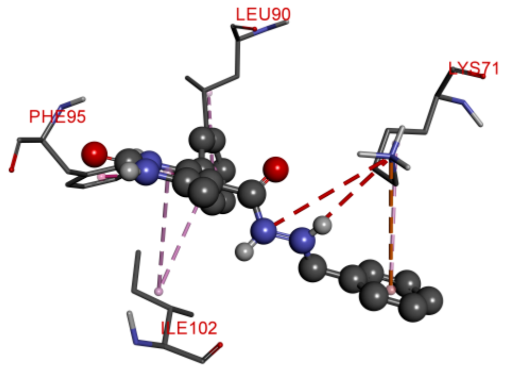	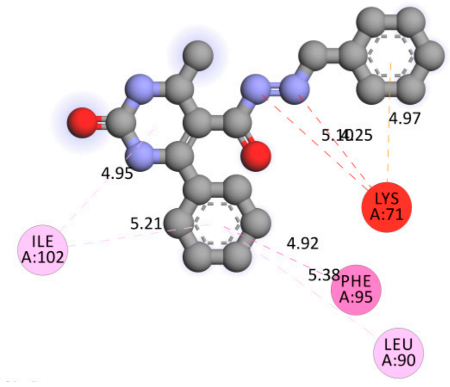
Compound **1c**	
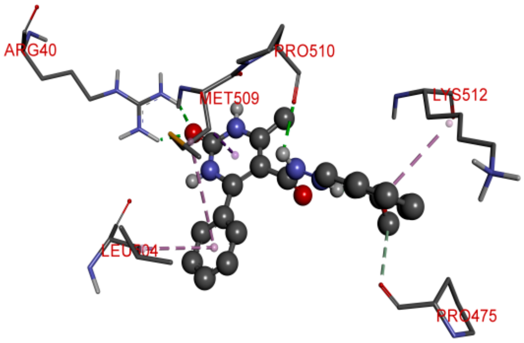	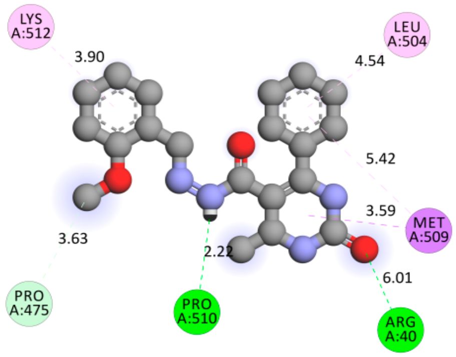
Compound **1d**	
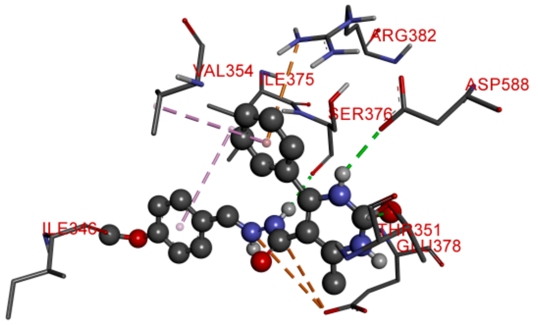	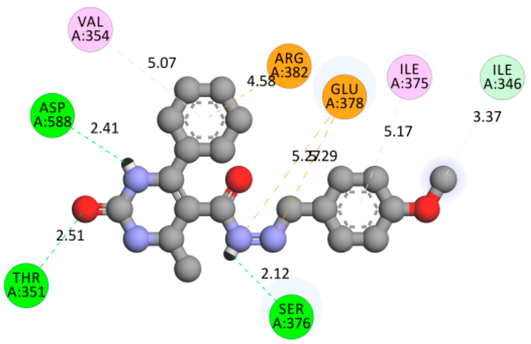
Compound **2a**	

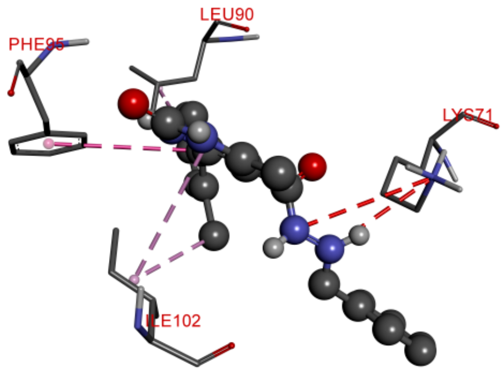

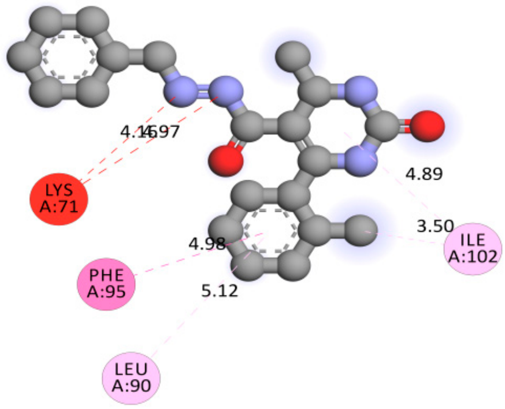

Compound **2c**

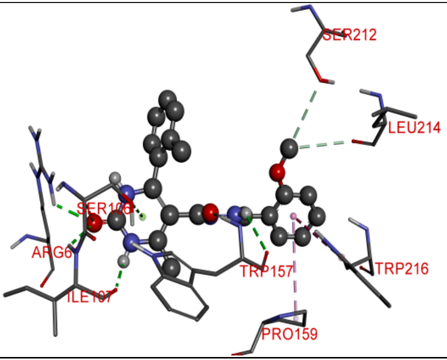

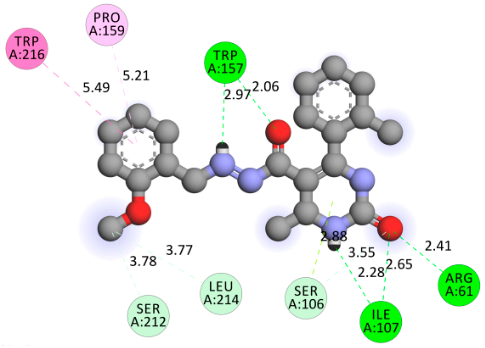

Compound **2d**

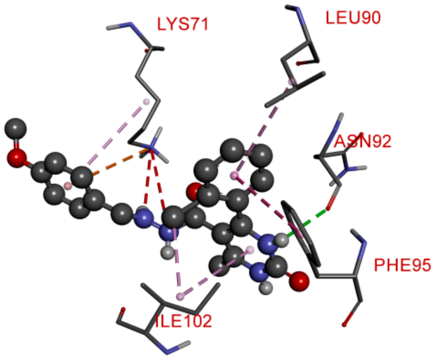

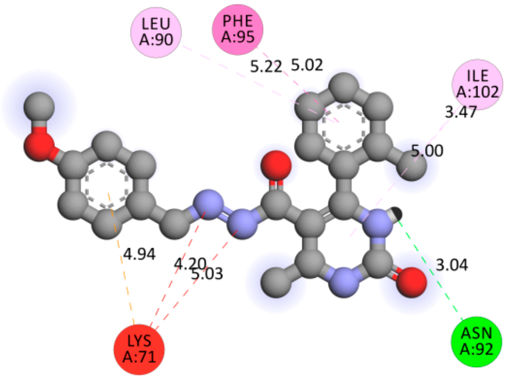

Compound **3a**

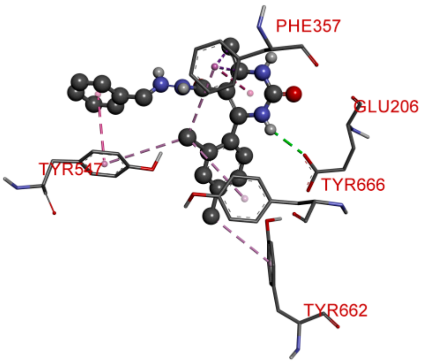

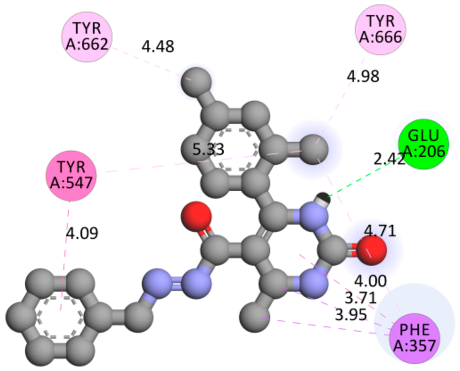

Compound **3c**

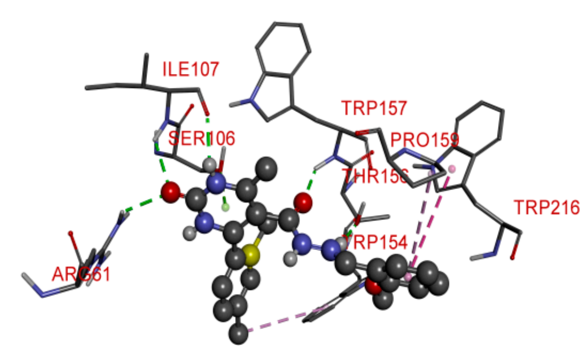

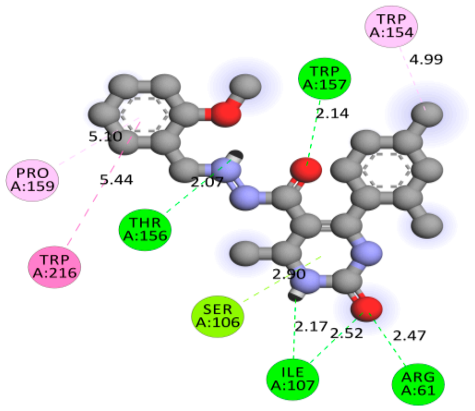

Compound **3d**

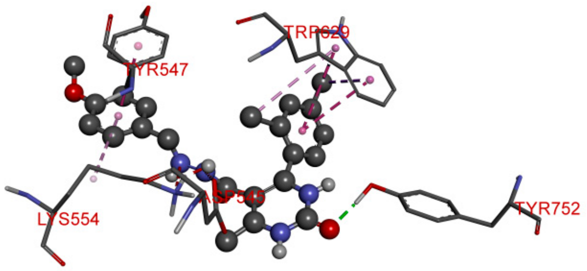

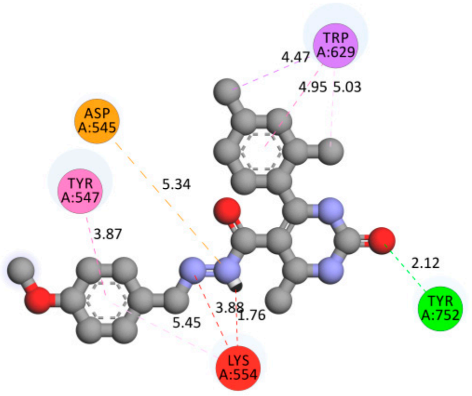

Compound **4b**

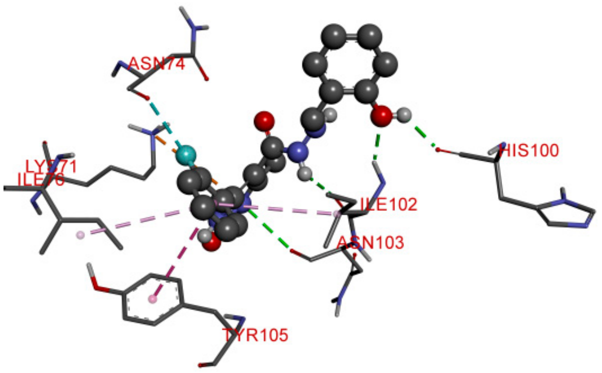

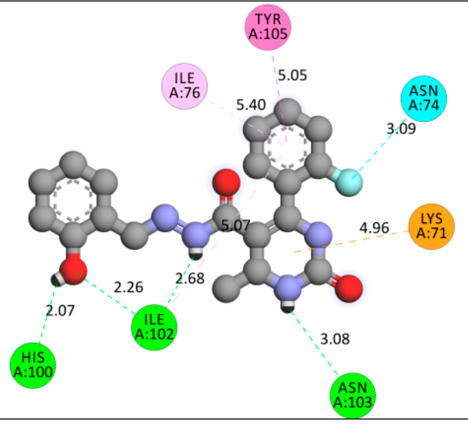

Compound **4c**

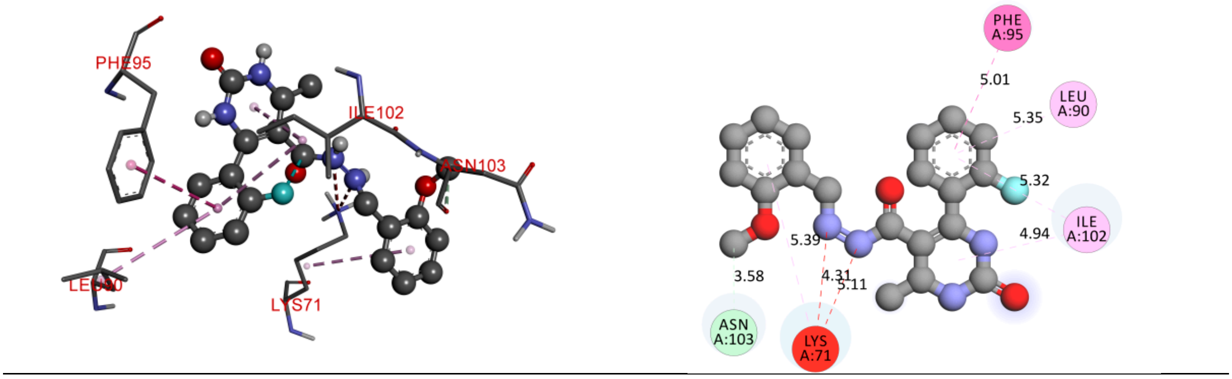

Compound **4d**

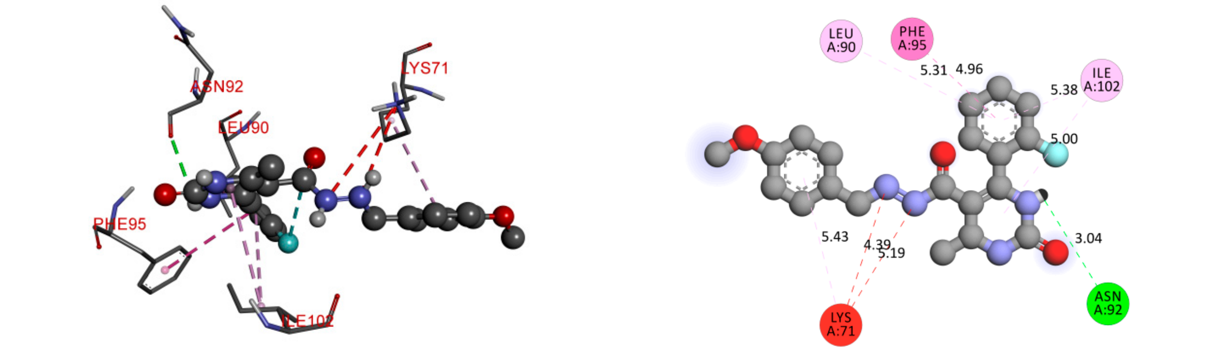

Compound **5a**

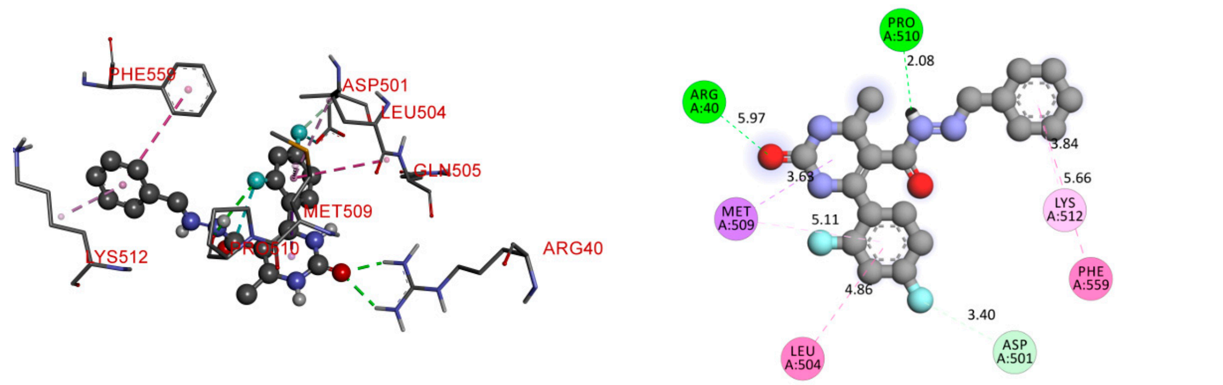

Compound **5c**

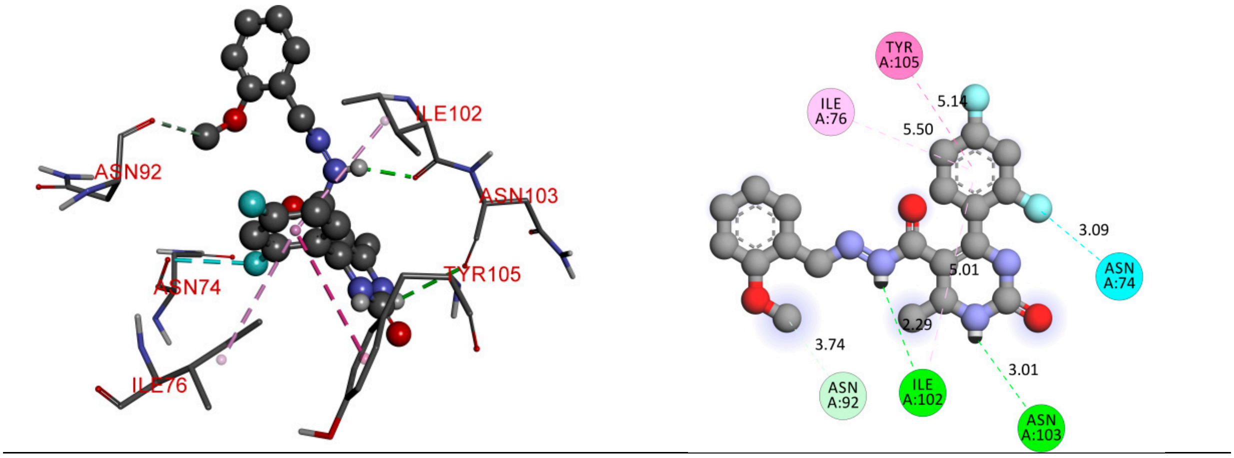

Compound **5d**	
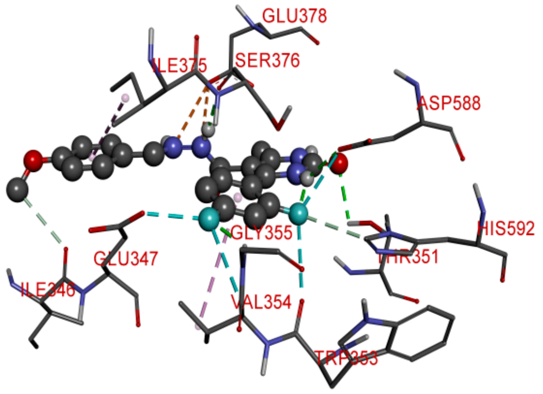GLU378<br>ILE375<br>SER376<br>ASP588<br>HIS592<br>GLY355<br>THR351<br>GLU347<br>ILE346<br>VAL354<br>TRP353	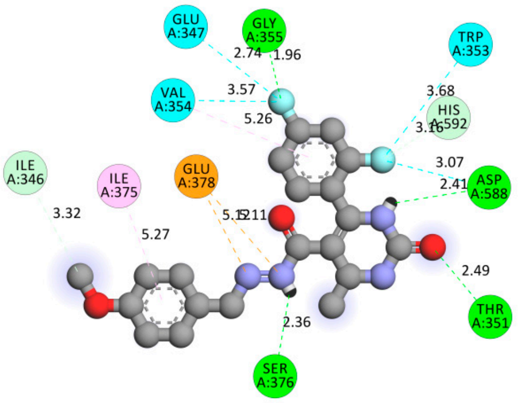GLU A:347<br>GLY A:355<br>TRP A:353<br>VAL A:354<br>HIS A:592<br>ASP A:588<br>ILE A:346<br>ILE A:375<br>GLU A:378<br>THR A:351<br>SER A:376<br>2.74<br>1.96<br>3.57<br>5.26<br>3.68<br>3.07<br>2.41<br>3.32<br>5.27<br>2.49<br>2.36
